# Supplementary material for: The Effect of Intraocular Pressure-Lowering Medication on Metastatic Uveal Melanomas
Source: Cancers (Basel). 2021 Nov 12;13(22):5657. doi: 10.3390/cancers13225657 (PMC8616129; doi:10.3390/cancers13225657)

Supplementary appendix

The effect of intraocular pressure-lowering medication on metastatic uveal melanomas

Contents

TABLE S1: Baseline characteristics of choroidal melanoma patients for those using prostaglandins/pilocarpines and controls included from 1996 onwards. Presented as mean±standard deviation unless stated otherwise.

TABLE S2: Baseline characteristics of choroidal melanoma patients for those using beta-blockers and controls included from 1986 onwards. Presented as mean±standard deviation unless stated otherwise.

TABLE S3: Baseline characteristics of choroidal melanoma patients for those using carbonic anhydrase inhibitors and controls included from 1994 onwards. Presented as mean±standard deviation unless stated otherwise.

TABLE S4: Baseline characteristics of choroidal melanoma patients for those using oral intraocular pressure-lowering medication and controls included from 1986 onwards. Presented as mean±standard deviation unless stated otherwise.

TABLE S5: Baseline characteristics of uveal melanoma patients for those using prostaglandins/pilocarpines and controls included from 1996 onwards. Presented as mean±standard deviation unless stated otherwise.

TABLE S6: Baseline characteristics of uveal melanoma patients for those using alpha2-agonists and controls included from 2000 onwards. Presented as mean±standard deviation unless stated otherwise.

TABLE S7: Baseline characteristics of uveal melanoma patients for those using beta-blockers and controls included from 1986 onwards. Presented as mean±standard deviation unless stated otherwise.

TABLE S8: Baseline characteristics of uveal melanoma patients for those using carbonic anhydrase inhibitors and controls included from 1994 onwards. Presented as mean±standard deviation unless stated otherwise.

TABLE S9: Baseline characteristics of uveal melanoma patients for those using oral intraocular pressure-lowering medication and controls included from 1986 onwards. Presented as mean±standard deviation unless stated otherwise.

TABLE S10: Risk of metastatic choroidal- and uveal melanoma and mortality in patients with a BAP1-mutation for those using prostaglandins/pilocarpines and controls included from 1996 onwards. Presented as hazard ratio with corresponding 95% confidence interval and p-value.

FIGURE S1: Kaplan-Meier cumulative incidence curve of choroidal- and ciliary body melanoma patients for metastasis (A) and mortality (B).

FIGURE S2: Kaplan-Meier cumulative incidence curves of metastasis (left) and mortality (right) in choroidal melanoma patients with and without prostaglandin/pilocarpine use (A and B), with and without beta-blocker use (C and D), with and without carbonic anhydrase inhibitor use (E and F), and with and without oral intraocular pressure-lowering medication use (G and H).

TABLE S1. Baseline characteristics of choroidal melanoma patients for those using prostaglandins/pilocarpines and controls included from 1996 onwards. Presented as mean±standard deviation unless stated otherwise.

	Prostaglandin/pilocarpine use (N= 6)	No Prostaglandin/pilocarpine use (N= 515)	p-value
Age (years)	67.9±15.8	62.9±12.9	0.520
Gender, male (N, %)	4 (53.0)	273 (66.7)	0.689
Caucasian descent (N, %)	6 (100.0)	507 (98.6)	1.000
IOP (mmHg)	22.7±19.5	15.2±6.2	0.401
IOP eye contralateral (mmHg)	13.8±2.6	15.6±3.4	0.190
Visual acuity	0.41±0.43	0.51±0.38	0.494
Spherical equivalent (D)	-0.63±2.63	0.59±2.66	0.436
IOP-lowering medication (N, %)			<0.001*
- Beta-blockers	2 (33.3)	7 (1.4)	0.004*
- CAI	2 (33.3)	7 (1.4)	0.004*
- Alpha-agonists	0 (0.0)	3 (0.6)	1.000
- Oral medication	1 (16.7)	4 (0.8)	0.056
TNM classification (N, %)			0.122
- T1	2 (33.3)	81 (15.8)	0.247
- T2	0 (0.0)	203 (39.6)	0.086
- T3	4 (66.7)	204 (39.8)	0.225
- T4	0 (0.0)	24 (4.7)	1.000
BAP1-mutations (N, %)$	4 (100.0)	299 (65.7)	0.305
SF3B1-mutations (N, %)$	0 (0.0)	50 (23.7)	NA
Treatment (N, %)			1.000
- Enucleation	4 (66.7)	303 (59.2)	1.000
- SRT	2 (33.3)	208 (40.6)	1.000
- Brachytherapy	0 (0.0)	0 (0.0)	NA
- Proton	0 (0.0)	1 (0.2)	1.000
- TTT	0 (0.0)	0 (0.0)	NA
- Local resection	0 (0.0)	0 (0.0)	NA
- Exenteration	0 (0.0)	0 (0.0)	NA
- PDT	0 (0.0)	0 (0.0)	NA
Malignancy in history (N, %)#	0 (0.0)	74 (14.4)	0.601
Metastatic diseases in history (N, %)#	0 (0.0)	8 (1.6)	1.000
Positive family history for UM (N, %)	0 (0.0)	8 (1.6)	1.000
Previous eye surgery (N, %)	2 (33.3)	62 (12.0)	0.161
Diabetes (N, %)	0 (0.0)	54 (10.5)	1.000
Hypertension (N, %)	1 (16.7)	168 (32.8)	0.669

*= Indicate statistical significance ($P < 0.05$); NA = not applicable/available; $ = Only patients with available tumor tissue; # = Excluding choroidal melanoma; IOP = Intraocular pressure; CAI = Carbonic anhydrase inhibitors; Oral medication = Acetazolamide; SRT = Stereotactic radiotherapy; TTT = Transpupillary Thermotherapy; PDT = Photodynamic therapy; UM = uveal melanoma

TABLE S2. Baseline characteristics of choroidal melanoma patients for those using beta-blockers and controls included from 1986 onwards. Presented as mean±standard deviation unless stated otherwise.

	Beta-blocker use (N= 10)	No Beta-blocker use (N= 560)	p-value
Age (years)	63.1±11.1	62.5±13.2	0.972
Gender, male (N, %)	7 (70.0)	300 (53.6)	0.354
Caucasian descent (N, %)	10 (100.0)	551 (98.6)	1.000
IOP (mmHg)	34.9±20.5	14.9±5.3	0.002*
IOP eye contralateral (mmHg)	13.4±2.4	15.6±3.4	0.031*
Visual acuity	0.12±0.16	0.51±0.38	<0.001*
Spherical equivalent (D)	-0.63±2.91	0.61±2.63	0.397
IOP-lowering medication (N, %)			<0.001*
- Prostaglandin/pilocarpine	2 (20.0)	4 (0.7)	0.004*
- CAI	7 (70.0)	2 (0.4)	<0.001*
- Alpha-agonists	3 (30.0)	0 (0.0)	<0.001*
- Oral medication	6 (60.0)	1 (0.2)	<0.001*
TNM classification (N, %)			0.364
- T1	2 (20.0)	100 (18.0)	0.698
- T2	2 (20.0)	221 (39.7)	0.329
- T3	5 (50.0)	210 (35.4)	0.515
- T4	1 (10.0)	26 (4.7)	0.389
BAP1 mutations (N, %)$	5 (62.5)	332 (66.4)	1.000
SF3B1 mutations (N, %)$	0 (0.0)	53 (24.0)	0.575
Treatment (N, %)			0.382
- Enucleation	8 (80.0)	348 (62.5)	0.336
- SRT	2 (20.0)	208 (37.3)	0.337
- Brachytherapy	0 (0.0)	0 (0.0)	NA
- Proton	0 (0.0)	1 (0.2)	1.000
- TTT	0 (0.0)	0 (0.0)	NA
- Local resection	0 (0.0)	0 (0.0)	NA
- Exenteration	0 (0.0)	0 (0.0)	NA
- PDT	0 (0.0)	0 (0.0)	NA
Malignancy in history (N, %)#	1 (10.0)	77 (13.8)	1.000
Metastatic diseases in history (N, %)#	0 (0.0)	8 (1.4)	1.000
Positive family history for UM (N, %)	0 (0.0)	8 (1.4)	1.000
Previous eye surgery (N, %)	2 (20.0)	63 (11.3)	0.319
Diabetes (N, %)	1 (10.0)	56 (10.1)	1.000
Hypertension (N, %)	2 (20.0)	172 (30.9)	0.731

*= Indicate statistical significance (P < 0.05); NA = not applicable/available; $ = Only patients with available tumor tissue; # = Excluding choroidal melanoma; IOP = Intraocular pressure; CAI = Carbonic anhydrase inhibitors; Oral medication = acetazolamide; SRT = Stereotactic radiotherapy; TTT = Transpupillary Thermotherapy; PDT = Photodynamic therapy; UM = Uveal melanoma

TABLE S3. Baseline characteristics of choroidal melanoma patients for those using carbonic anhydrase inhibitors and controls included from 1994 onwards. Presented as mean±standard deviation unless stated otherwise.

	CAI use (N= 9)	No CAI use (N= 534)	p-value
Age (years)	61.7±12.0	62.8±13.2	0.861
Gender, male (N, %)	6 (66.7)	284 (53.2)	0.513
Caucasian descent (N, %)	9 (100.0)	525 (98.5)	1.000
IOP (mmHg)	30.2±17.6	14.9±5.3	0.006*
IOP eye contralateral (mmHg)	13.1±2.1	15.7±3.4	0.027
Visual acuity	0.12±0.16	0.51±0.38	0.001*
Spherical equivalent (D)	0.54±2.06	0.62±2.64	0.858
IOP-lowering medication (N, %)			<0.001*
- Prostaglandin/pilocarpine	2 (22.2)	3 (0.6)	0.002*
- Beta-blockers	7 (77.8)	1 (0.2)	<0.001*
- Alpha-agonists	3 (33.3)	0 (0.0)	<0.001*
- Oral medication	4 (44.4)	1 (0.2)	<0.001*
TNM classification (N, %)			0.109
- T1	3 (33.3)	88 (16.6)	0.181
- T2	1 (11.1)	212 (39.9)	0.096
- T3	4 (44.4)	207 (39.0)	0.742
- T4	1 (11.1)	24 (4.5)	0.349
BAP1 mutations (N, %)$	3 (42.9)	313 (66.0)	0.239
SF3B1 mutations (N, %)$	0 (0.0)	52 (23.7)	0.593
Treatment (N, %)			0.015*
- Enucleation	6 (66.7)	323 (60.8)	1.000
- SRT	2 (22.2)	208 (39.2)	0.493
- Brachytherapy	0 (0.0)	0 (0.0)	NA
- Proton	1 (11.1)	0 (0.0)	0.017*
- TTT	0 (0.0)	0 (0.0)	NA
- Local resection	0 (0.0)	0 (0.0)	NA
- Exenteration	0 (0.0)	0 (0.0)	NA
- PDT	0 (0.0)	0 (0.0)	NA
Malignancy in history (N, %)#	0 (0.0)	77 (14.4)	0.372
Metastatic diseases in history (N, %)#	0 (0.0)	8 (1.5)	1.000
Positive family history for UM (N, %)	0 (0.0)	8 (1.5)	1.000
Previous eye surgery (N, %)	0 (0.0)	64 (12.0)	0.608
Diabetes (N, %)	2 (22.2)	53 (10.0)	0.232
Hypertension (N, %)	1 (11.1)	173 (32.6)	0.283

*= Indicate statistical significance (P < 0.05); NA = not applicable/available; $ = Only patients with available tumor tissue; # = Excluding choroidal melanoma; IOP = Intraocularpressure; CAI = Carbonic anhydrase inhibitors; Oral medication = acetazolamide; SRT = Stereotactic radiotherapy; TTT = Transpupillary Thermotherapy; PDT = Photodynamic therapy; UM = Uveal melanoma

TABLE S4. Baseline characteristics of choroidal melanoma patients for those using oral intraocular pressure-lowering medication and controls included from 1986 onwards. Presented as mean±standard deviation unless stated otherwise.

	Oral medication use (N= 7)	No oral medication use (N= 563)	p-value
Age (years)	58.2±10.9	62.5±13.2	0.314
Gender, male (N, %)	6 (85.7)	301 (53.5)	0.131
Caucasian descent (N, %)	7 (100.0)	554 (98.6)	1.000
IOP (mmHg)	44.3±16.9	14.9±5.3	<0.001*
IOP eye contralateral (mmHg)	13.5±2.4	15.6±3.4	0.102
Visual acuity	0.09±0.24	0.51±0.38	0.001*
Spherical equivalent (D)	0.00	0.60±2.64	0.683
IOP-lowering medication (N, %)			<0.001*
- Prostaglandin/pilocarpine	1 (14.3)	5 (0.9)	0.072
- Beta-blockers	6 (85.7)	4 (0.7)	<0.001*
- CAI	4 (57.1)	5 (0.9)	<0.001*
- Alpha-agonists	3 (42.9)	0 (0.0)	<0.001*
TNM classification (N, %)			0.850
- T1	1 (14.3)	101 (18.0)	1.000
- T2	2 (28.6)	221 (39.5)	0.710
- T3	4 (57.1)	211 (37.7)	0.435
- T4	0 (0.0)	27 (4.8)	1.000
BAP1 mutations (N, %)$	5 (71.4)	332 (66.3)	1.000
SF3B1 mutations (N, %)$	0 (0.0)	53 (23.9)	1.000
Treatment (N, %)			0.096
- Enucleation	7 (100.0)	349 (62.3)	0.050
- SRT	0 (0.0)	210 (37.5)	0.050
- Brachytherapy	0 (0.0)	0 (0.0)	NA
- Proton	0 (0.0)	1 (0.2)	1.000
- TTT	0 (0.0)	0 (0.0)	NA
- Local resection	0 (0.0)	0 (0.0)	NA
- Exenteration	0 (0.0)	0 (0.0)	NA
- PDT	0 (0.0)	0 (0.0)	NA
Malignancy in history (N, %)#	0 (0.0)	78 (13.9)	0.601
Metastatic diseases in history (N, %)#	0 (0.0)	8 (1.4)	1.000
Positive family history for UM(N, %)	0 (0.0)	8 (1.4)	1.000
Previous eye surgery (N, %)	1 (14.3)	64 (11.4)	0.574
Diabetes (N, %)	0 (0.0)	57 (10.2)	1.000
Hypertension (N, %)	0 (0.0)	174 (31.1)	0.106

*= Indicate statistical significance (P<0.05); NA = not applicable/available; $ = Only patients with available tumor tissue; # = Excluding choroidal melanoma; IOP = Intraocular pressure; CAI = Carbonic anhydrase inhibitors; Oral medication = acetazolamide; SRT = Stereotactic radiotherapy; TTT = Transpupillary Thermotherapy; PDT = Photodynamic therapy; UM = uveal melanoma

TABLE S5. Baseline characteristics of uveal melanoma patients for those using prostaglandins/pilocarpines and controls included from 1996 onwards. Presented as mean±standard deviation unless stated otherwise.

	Prostaglandin/pilocarpine use (N= 13)	No Prostaglandin/pilocarpine use (N= 629)	p-value
Age (years)	67.2±13.7	63.4±12.6	0.454
Gender, male (N, %)	9 (69.2)	312 (49.6)	0.161
Caucasian descent (N, %)	13 (100.0)	621 (98.9)	1.000
IOP (mmHg)	22.2±14.0	15.4±6.2	0.020*
IOP eye contralateral (mmHg)	14.5±2.6	15.7±3.5	0.208
Visual acuity	0.30±0.34	0.52±0.38	0.034*
Spherical equivalent (D)	-0.66±1.91	0.52±2.64	0.153
Diagnosis (N, %)			0.016*
- Choroidal melanoma	6 (60.0)	515 (89.7)	0.016*
- Ciliary body melanoma	4 (40.0)	59 (10.3)	0.016*
IOP-lowering medication (N, %)			<0.001*
- Beta-blockers	5 (38.5)	8 (1.3)	<0.001*
- CAI	4 (30.8)	7 (1.1)	<0.001*
- Alpha-agonists	1 (7.7)	3 (0.5)	0.079
- Oral medication	5 (38.5)	5 (0.8)	<0.001*
TNM classification (N, %)			0.531
- T1	3 (25.0)	108 (17.5)	0.453
- T2	3 (25.0)	235 (38.1)	0.550
- T3	5 (41.7)	239 (38.8)	1.000
- T4	1 (8.3)	34 (5.5)	0.501
BAP1-mutations (N, %)$	7 (77.8)	339 (66.0)	0.725
SF3B1-mutations (N, %)$	1 (33.3)	58 (24.1)	0.566
Treatment (N, %)			0.083
- Enucleation	7 (53.8)	341 (55.0)	0.934
- SRT	3 (23.1)	240 (38.7)	0.389
- Brachytherapy	2 (15.4)	15 (2.4)	0.045*
- Proton	1 (7.7)	5 (0.8)	0.118
- TTT	0 (0.0)	10 (1.6)	1.000
- Local resection	0 (0.0)	3 (0.5)	1.000
- Exenteration	0 (0.0)	1 (0.2)	1.000
- PDT	0 (0.0)	2 (0.3)	1.000
Malignancy in history (N, %)#	0 (0.0)	94 (14.9)	0.233
Metastatic diseases in history (N, %)#	0 (0.0)	9 (1.4)	1.000
Positive family history for UM (N, %)	0 (0.0)	9 (1.4)	1.000
Previous eye surgery (N, %)	3 (23.1)	88 (14.0)	0.410
Diabetes (N, %)	1 (7.7)	62 (9.9)	1.000
Hypertension (N, %)	3 (23.1)	205 (32.8)	0.562

*= Indicate statistical significance ($P < 0.05$); $ = Only patients with available tumor tissue; # = Excluding uveal melanoma; IOP = Intraocular pressure; CAI = Carbonic anhydrase inhibitors; Oral medication = Acetazolamide; SRT = Stereotactic radiotherapy; TTT = Transpupillary Thermotherapy; PDT = Photodynamic therapy; UM = Uveal melanoma

TABLE S6. Baseline characteristics of uveal melanoma patients for those using alpha2-agonists and controls included from 2000 onwards. Presented as mean±standard deviation unless stated otherwise.

	Alpha-agonist use (N= 4)	No Alpha-agonist use (N= 585)	p-value
Age (years)	64.5±10.6	63.6±12.5	0.881
Gender, male (N, %)	2 (50.0)	293 (50.1)	1.000
Caucasian descent (N, %)	4 (100.0)	577 (98.8)	1.000
IOP (mmHg)	41.5±14.6	15.3±5.5	0.001*
IOP eye contralateral (mmHg)	14.0±1.0	15.7±3.5	0.307
Visual acuity	0.14±0.26	0.54±0.38	0.020*
Spherical equivalent (D)	-3.13	0.51±2.62	0.182
Diagnosis (N, %)			0.374
- Choroidal melanoma	3 (75.0)	474 (89.1)	0.374
- Ciliary body melanoma	1 (25.0)	58 (10.9)	0.374
IOP-lowering medication (N, %)			<0.001*
- Prostaglandin/pilocarpine	1 (25.0)	11 (1.9)	0.079
- Beta-blockers	4 (100.0)	8 (1.4)	<0.001*
- CAI	4 (100.0)	7 (1.2)	<0.001*
- Oral medication	4 (100.0)	5 (0.8)	<0.001*
TNM classification (N, %)			1.000
- T1	0 (0.0)	102 (17.8)	1.000
- T2	2 (50.0)	216 (37.7)	0.635
- T3	2 (50.0)	222 (38.7)	0.644
- T4	0 (0.0)	33 (5.8)	1.000
BAP1 mutations (N, %)$	2 (50.0)	321 (68.3)	0.595
SF3B1 mutations (N, %)$	1 (33.3)	47 (23.6)	0.559
Treatment (N, %)			0.374
- Enucleation	4 (100.0)	297 (52.4)	0.125
- SRT	0 (0.0)	242 (41.9)	0.145
- Brachytherapy	0 (0.0)	17 (2.9)	1.000
- Proton	0 (0.0)	6 (1.0)	1.000
- TTT	0 (0.0)	7 (1.2)	1.000
- Local resection	0 (0.0)	2 (0.3)	1.000
- Exenteration	0 (0.0)	1 (0.2)	1.000
- PDT	0 (0.0)	2 (0.3)	1.000
Malignancy in history (N, %)#	0 (0.0)	88 (15.0)	1.000
Metastatic diseases in history (N, %)#	0 (0.0)	7 (1.2)	1.000
Positive family history for UM (N, %)	0 (0.0)	9 (1.5)	1.000
Previous eye surgery (N, %)	0 (0.0)	86 (14.7)	1.000
Diabetes (N, %)	0 (0.0)	61 (10.4)	1.000
Hypertension (N, %)	1 (20.0)	194 (32.9)	0.308

*= Indicate statistical significance (P < 0.05); $ = Only patients with available tumor tissue; # = Excluding uveal melanoma; IOP = Intraocular pressure; CAI = Carbonic anhydrase inhibitors; Oral medication = acetazolamide; SRT = Stereotactic radiotherapy; TTT = Transpupillary Thermotherapy; PDT = Photodynamic therapy; UM = Uveal melanoma

TABLE S7. Baseline characteristics of uveal melanoma patients for those using beta-blockers and controls included from 1986 onwards. Presented as mean±standard deviation unless stated otherwise.

	Beta-blocker use (N= 14)	No Beta-blocker use (N= 693)	p-value
Age (years)	60.7±10.4	63.1±13.1	0.343
Gender, male (N, %)	9 (64.3)	350 (50.5)	0.420
Caucasian descent (N, %)	14 (100.0)	692 (98.8)	1.000
IOP (mmHg)	32.2±18.6	15.2±5.5	0.001*
IOP eye contralateral (mmHg)	14.2±2.5	15.7±3.5	0.106
Visual acuity	0.13±0.18	0.52±0.38	<0.001*
Spherical equivalent (D)	-1.00±2.39	0.52±2.61	0.142
Diagnosis (N, %)			0.170
- Choroidal melanoma	10 (76.9)	560 (89.0)	0.170
- Ciliary body melanoma	3 (23.1)	69 (11.0)	0.170
IOP-lowering medication (N, %)			<0.001*
- Prostaglandin/pilocarpine	5 (35.7)	8 (1.2)	<0.001*
- CAI	9 (64.3)	2 (0.3)	<0.001*
- Alpha-agonists	4 (28.6)	0 (0.0)	<0.001*
- Oral medication	10 (71.4)	2 (0.3)	<0.001*
TNM classification (N, %)			0.458
- T1	2 (15.4)	132 (19.5)	1.000
- T2	3 (23.1)	259 (38.3)	0.389
- T3	7 (53.8)	248 (36.7)	0.249
- T4	1 (7.7)	37 (5.5)	0.525
BAP1 mutations (N, %)$	7 (58.3)	380 (66.7)	0.548
SF3B1 mutations (N, %)$	1 (16.7)	63 (24.8)	1.000
Treatment (N, %)			0.349
- Enucleation	10 (71.4)	698 (58.2)	0.416
- SRT	3 (21.4)	240 (35.1)	0.400
- Brachytherapy	0 (0.0)	21 (3.1)	1.000
- Proton	1 (7.1)	5 (0.7)	0.115
- TTT	0 (0.0)	11 (1.6)	1.000
- Local resection	0 (0.0)	3 (0.4)	1.000
- Exenteration	0 (0.0)	1 (0.1)	1.000
- PDT	0 (0.0)	2 (0.3)	1.000
Malignancy in history (N, %)#	1 (7.1)	98 (14.1)	0.705
Metastatic diseases in history (N, %)#	0 (0.0)	9 (1.3)	1.000
Positive family history for UM (N, %)	0 (0.0)	9 (1.3)	1.000
Previous eye surgery (N, %)	2 (14.3)	94 (13.6)	1.000
Diabetes (N, %)	1 (7.1)	65 (9.5)	1.000
Hypertension (N, %)	2 (14.3)	213 (31.0)	0.247

*= Indicate statistical significance (P < 0.05); $ = Only patients with available tumor tissue; # = Excluding uveal melanoma; IOP = Intraocular pressure; CAI = Carbonic anhydrase inhibitors; Oral medication = acetazolamide; SRT = Stereotactic radiotherapy; TTT = Transpupillary Thermotherapy; PDT = Photodynamic therapy; UM = Uveal melanoma

TABLE S8. Baseline characteristics of uveal melanoma patients for those using carbonic anhydrase inhibitors and controls included from 1994 onwards. Presented as mean±standard deviation unless stated otherwise.

	CAI use (N= 11)	No CAI use (N= 658)	p-value
Age (years)	60.2±11.4	63.3±13.0	0.424
Gender, male (N, %)	7 (63.6)	330 (50.2)	0.375
Caucasian descent (N, %)	11 (100.0)	649 (98.8)	1.000
IOP (mmHg)	28.7±17.1	15.2±5.5	0.016*
IOP eye contralateral (mmHg)	13.7±2.1	15.7±3.5	0.054
Visual acuity	0.15±0.19	0.52±0.38	0.001*
Spherical equivalent (D)	-0.30±2.16	0.52±2.62	0.361
Diagnosis (N, %)			1.000
- Choroidal melanoma	9 (90.0)	534 (89.3)	1.000
- Ciliary body melanoma	1 (10.0)	64 (10.7)	1.000
IOP-lowering medication (N, %)			<0.001*
- Prostaglandin/pilocarpine	4 (36.4)	8 (1.2)	<0.001*
- Beta-blockers	9 (18.8)	3 (0.5)	<0.001*
- Alpha-agonists	4 (36.4)	0 (0.0)	<0.001*
- Oral medication	6 (54.5)	4 (0.6)	<0.001*
TNM classification (N, %)			0.362
- T1	3 (30.0)	116 (18.0)	0.400
- T2	2 (20.0)	249 (38.7)	0.331
- T3	4 (40.0)	244 (37.9)	1.000
- T4	1 (10.0)	35 (5.4)	0.435
BAP1 mutations (N, %)$	3 (33.3)	356 (66.2)	0.070
SF3B1 mutations (N, %)$	1 (16.7)	62 (24.6)	1.000
Treatment (N, %)			0.043*
- Enucleation	7 (63.6)	365 (56.2)	0.764
- SRT	2 (18.2)	241 (37.1)	0.344
- Brachytherapy	0 (0.0)	20 (3.1)	1.000
- Proton	2 (18.2)	4 (0.6)	0.004*
- TTT	0 (0.0)	10 (1.5)	1.000
- Local resection	0 (0.0)	3 (0.5)	1.000
- Exenteration	0 (0.0)	1 (0.2)	1.000
- PDT	0 (0.0)	2 (0.3)	1.000
Malignancy in history (N, %)#	0 (0.0)	97 (14.7)	0.381
Metastatic diseases in history (N, %)#	0 (0.0)	9 (1.4)	1.000
Positive family history for UM (N, %)	0 (0.0)	9 (1.3)	1.000
Previous eye surgery (N, %)	0 (0.0)	93 (14.1)	0.377
Diabetes (N, %)	2 (18.2)	62 (9.5)	0.288
Hypertension (N, %)	1 (9.1)	212 (32.5)	0.116

*= Indicate statistical significance ($P < 0.05$); $ = Only patients with available tumor tissue; # = Excluding uveal melanoma; IOP = Intraocular pressure; CAI = Carbonic anhydrase inhibitors; Oral medication = acetazolamide; SRT = Stereotactic radiotherapy; TTT = Transpupillary Thermotherapy; PDT = Photodynamic therapy; UM = Uveal melanoma

TABLE S9. Baseline characteristics of uveal melanoma patients for those using oral intraocular pressure-lowering medication and controls included from 1986 onwards. Presented as mean±standard deviation unless stated otherwise.

	Oral medication use (N= 12)	No oral medication use (N= 695)	p-value
Age (years)	58.1±9.7	63.1±13.1	0.115
Gender, male (N, %)	9 (75.0)	352 (50.4)	0.090
Caucasian descent (N, %)	12 (100.0)	686 (98.8)	1.000
IOP (mmHg)	36.8±16.9	15.2±5.5	<0.001*
IOP eye contralateral (mmHg)	14.3±2.5	15.7±3.5	0.166
Visual acuity	0.10±0.22	0.52±0.38	<0.001*
Spherical equivalent (D)	-1.04±1.80	0.51±2.61	0.176
Diagnosis (N, %)			0.026*
- Choroidal melanoma	7 (63.6)	563 (89.2)	0.026*
- Ciliary body melanoma	4 (36.4)	68 (10.8)	0.026*
IOP-lowering medication (N, %)			<0.001*
- Prostaglandin/pilocarpine	5 (41.7)	8 (1.2)	<0.001*
- Beta-blockers	10 (83.3)	4 (0.6)	<0.001*
- CAI	6 (50.0)	5 (0.7)	<0.001*
- Alpha-agonists	4 (33.3)	0 (0.0)	<0.001*
TNM classification (N, %)			0.699
- T1	1 (9.1)	133 (19.6)	0.701
- T2	4 (36.4)	258 (38.1)	1.000
- T3	6 (54.5)	249 (36.7)	0.345
- T4	0 (0.0)	38 (5.6)	1.000
BAP1 mutations (N, %)$	8 (66.7)	379 (66.5)	1.000
SF3B1 mutations (N, %)$	1 (20.0)	63 (24.7)	1.000
Treatment (N, %)			0.144
- Enucleation	10 (83.3)	398 (58.0)	0.136
- SRT	1 (8.3)	242 (35.3)	0.066
- Brachytherapy	0 (0.0)	21 (3.1)	1.000
- Proton	1 (8.3)	5 (0.7)	0.099
- TTT	0 (0.0)	11 (1.6)	1.000
- Local resection	0 (0.0)	3 (0.4)	1.000
- Exenteration	0 (0.0)	1 (0.1)	1.000
- PDT	0 (0.0)	2 (0.3)	1.000
Malignancy in history (N, %)#	0 (0.0)	99 (14.2)	0.390
Metastatic diseases in history (N, %)#	0 (0.0)	9 (1.3)	1.000
Positive family history for UM(N, %)	0 (0.0)	9 (1.3)	1.000
Previous eye surgery (N, %)	2 (16.7)	94 (13.5)	0.672
Diabetes (N, %)	0 (0.0)	66 (9.6)	0.616
Hypertension (N, %)	1 (8.3)	214 (31.1)	0.118

*= Indicate statistical significance (P<0.05); $ = Only patients with available tumor tissue; # = Excluding uveal melanoma; IOP = Intraocular pressure; CAI = Carbonic anhydrase inhibitors; Oral medication = acetazolamide; SRT = Stereotactic radiotherapy; TTT = Transpupillary Thermotherapy; PDT = Photodynamic therapy; UM = Uveal melanoma

TABLE S10. Risk of metastatic choroidal- and uveal melanoma and mortality in patients with a *BAP1*-mutation for those using prostaglandins/pilocarpines and controls included from 1996 onwards. Presented as hazard ratio with corresponding 95% confidence interval and p-value.

	Risk of metastasis			Risk of mortality		
	Hazard ratio	95% confidence interval	p-value	Hazard ratio	95% confidence interval	p-value
CM$^{\alpha}$	16.868	3.698-76.946	p<0.001*	13.271	3.070-57.364	p=0.001*
UM (CB+CBM)$^{\beta}$	7.731	2.046-29.210	p=0.003*	9.794	2.391-40.123	p=0.002*

*= Indicate statistical significance (P < 0.05); α = Adjusted for age, gender, IOP, IOP-lowering medication and tumor treatment (Supplementary Table S3); β = Adjusted for age, gender, IOP, IOP-lowering medication, tumor diagnosis and tumor treatment (Supplementary Table S5); CM = Choroidal melanoma; CBM = Ciliary body melanoma; UM = Uveal melanoma

FIGURE S1. Kaplan-Meier cumulative incidence curve of choroidal- and ciliary body melanoma patients for metastasis (A) and mortality (B).

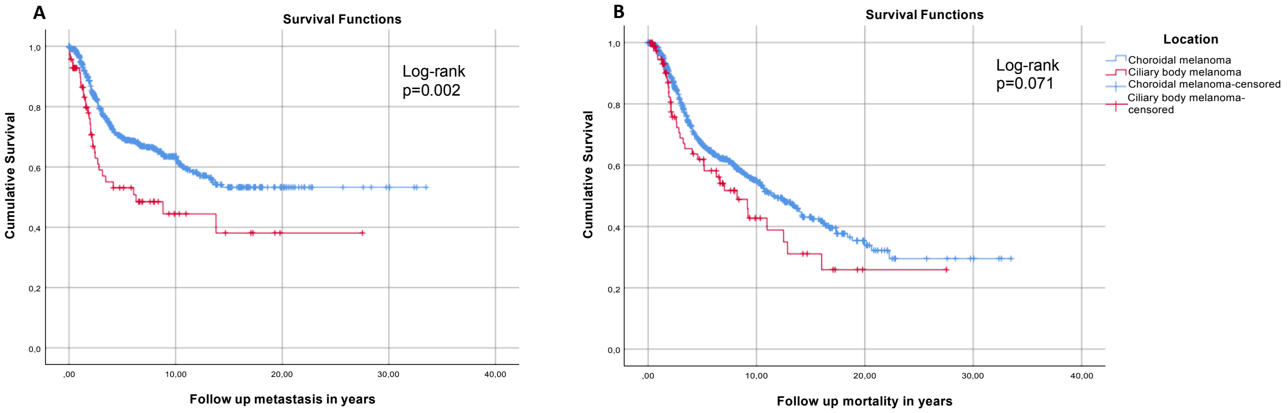

FIGURE S2. Kaplan-Meier cumulative incidence curves of metastasis (left) and mortality (right) in choroidal melanoma patients with and without prostaglandin/pilocarpine use (A and B), with and without beta-blocker use (C and D), with and without carbonic anhydrase inhibitor use (E and F), and with and without oral intraocular pressure-lowering medication use (G and H).

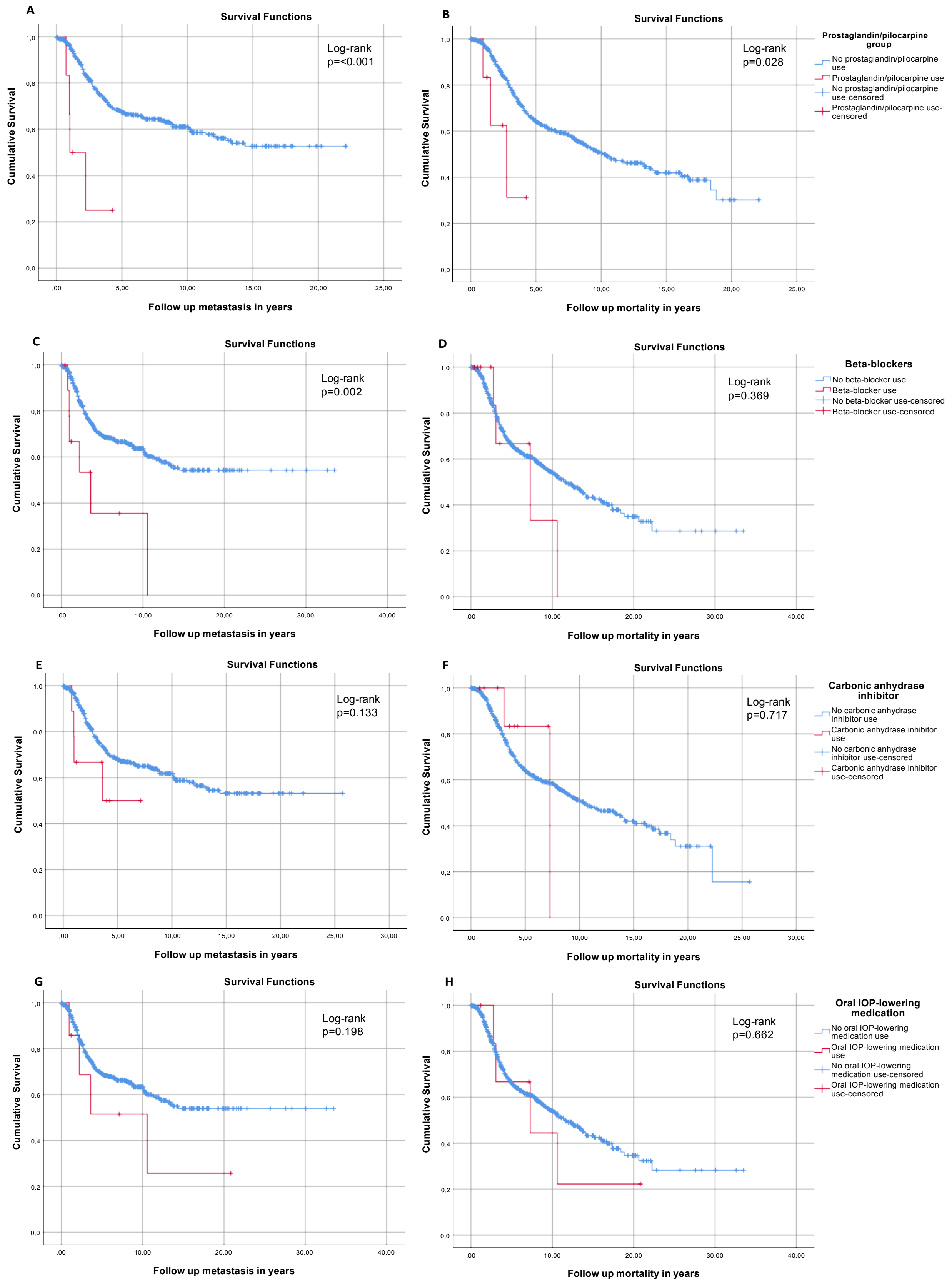

Supplement: Supplementary file 1 [file cancers-13-05657-s001.zip › cancers-1356370-supplementary.pdf]
